# Superb microvascular imaging within intestinal ultrasonography predicts endoscopic ulcers in the terminal ileum of patients with Crohn’s disease

**DOI:** 10.1097/MD.0000000000050365

**Published:** 2026-08-21

**Authors:** Masanao Nasuno, Airi Konno, Kazuki Shimoyama, Takahito Hamada, Kohei Sugiyama, Maki Miyakawa, Masanori Nojima, Hiroki Tanaka

**Affiliations:** a Sapporo IBD Clinic, Sapporo, Japan; b Department of Radiology, Sapporo IBD Clinic, Sapporo, Japan; c Department of Gastroenterology, Sapporo Central Hospital, Sapporo, Japan; d Center for Translational Research, Institute of Medical Science Hospital, University of Tokyo, Tokyo, Japan.

**Keywords:** Crohn’s disease, intestinal ultrasonography, superb microvascular imaging

## Abstract

This retrospective study aimed to investigate whether superb microvascular imaging (SMI) can predict endoscopic findings in the terminal ileum of patients with Crohn’s disease (CD). Patients with CD who underwent intestinal ultrasonography (IUS) between September 2020 and April 2024 and ileocolonoscopy for terminal ileum evaluation within 3 months before or after IUS were included. Those who previously underwent small bowel resection were excluded. Aplio a550 was used for IUS. SMI signals were evaluated using simplified SMI grades (0–3) and a modified Limberg score based on SMI (mLS-SMI, 0–4). Endoscopic findings were categorized as no, aphthous, small, or large ulcer. The proportion of patients with each endoscopic finding was determined according to mLS-SMI and SMI grades. Furthermore, the sensitivity and specificity of each grade for predicting corresponding endoscopic findings were compared. In total, 204 patients were included, with 96, 68, 25, and 15 having no, aphthous, small, and large ulcers, respectively. Additionally, 161, 14, 13, 11, and 5 patients had mLS-SMI grades 0, 1, 2, 3, and 4, while 157, 27, 12, and 8 patients had SMI grades 0, 1, 2, and 3, respectively. As the mLS-SMI and SMI grades increased, the proportion of patients with aphthous, small, or large ulcers also increased. Likewise, the proportion of patients with small or large ulcers increased. The sensitivity and specificity for predicting aphthous, small, or large ulcers were 24% and 97% for mLS-SMI grade ≥2, and 37% and 93% for SMI grade ≥1, respectively. For predicting small or large ulcers, they were 50% and 95% for mLS-SMI grade ≥2, and 70% and 88% for SMI grade ≥1, respectively. SMI grade ≥ 1 offered a significantly higher sensitivity for predicting small or large ulcers than mLS-SMI grade ≥ 2 (*P* = .008). Higher mLS-SMI and SMI grades corresponded to an increased prevalence of endoscopic ulcers. Therefore, SMI grading may provide additional information for evaluating ulcerative lesions in patients with CD.

## 1. Introduction

Crohn’s disease (CD) is a chronic inflammatory disease of the gastrointestinal tract characterized by relapsing and remitting symptoms, potentially leading to serious complications and disability if not optimally treated.^[1,2]^ Currently, ileocolonoscopy remains the gold standard for monitoring intestinal lesions in CD. However, frequent assessment using ileocolonoscopy is invasive. Consequently, less invasive imaging modalities, particularly intestinal ultrasonography (IUS), have been increasingly recognized as useful alternatives for assessing endoscopic activity.

IUS is a noninvasive, cost-effective, radiation-free, and easy-to-assess modality that can quickly generate results, enabling rapid clinical decision-making. It is also highly acceptable and preferred by patients with CD over other modalities.^[3]^ Meta-analyses have demonstrated that, compared with magnetic resonance enterography and computed tomography, IUS provides high and comparable sensitivity and specificity for the primary diagnosis, detection of complications, and follow-up of intestinal involvement in patients with CD.^[4,5]^ Current guidelines from the European Crohn’s and Colitis Organization (ECCO), European Society of Gastrointestinal and Abdominal Radiology, and European Federation of Societies for Ultrasound in Medicine and Biology have recognized IUS as a valid tool for diagnosing CD and for detecting relapse or monitoring response to medical treatment during follow-up.^[3,6]^ According to a recent multicenter study, response to therapy was associated with a statistically significant reduction in bowel wall thickness (BWT) or bowel wall stratification (BWS), decreased fibrofatty proliferation, and increased signals on color Doppler ultrasound.^[7]^ BWT is the most established parameter for assessing disease activity for CD and has low interobserver variability.^[8]^ Additionally, a semiquantitative scale of Limberg score correlates well with both the endoscopic activity and histological findings.^[9-11]^ However, the conventional Doppler technique has limitations in detecting small vessels and low blood flow velocity.^[12]^ Contrast-enhanced ultrasonography (CEUS) reportedly can increase sensitivity in detecting the vascularization of the intestinal wall compared with the color Doppler mode in various organs.^[13-15]^ Nevertheless, CEUS also has some limitations. For example, contrast agents are not approved for intestinal ultrasound in some countries; the high cost of the intravenous contrast agent makes repeated examinations in daily clinical practice difficult; and allergic reactions caused by such agents may occur.^[16]^

Superb microvascular imaging (SMI) is a novel ultrasound technique for assessing slow and microvascular blood flow.^[17]^ It uses a multidimensional filter to separate flow signals from clutter, making microvascular slow flow signals visible, and depicts these images at high frame rates. SMI is also more economical and safer, given that it does not require contrast agents. Recently, SMI has been reported to be more sensitive than conventional color Doppler ultrasound in imaging several organs, including the kidney, ovary, and thyroid.^[18-20]^ However, only a few studies have evaluated the use of SMI in assessing intestinal lesions in CD cases. We previously published the first report demonstrating the utility of SMI for evaluating the intestinal lesions of patients with CD, in which we investigated the serum levels of leucine-rich alpha-2 glycoprotein for predicting active IUS findings in such intestinal lesions during clinical remission.^[21]^ Subsequently, Haberkamp et al reported that their original SMI scoring system applied in 56 patients showed a higher correlation with the segmental simple endoscopic score for Crohn’s disease than Doppler-based imaging scores.^[22]^ However, no large-scale studies have reported the use of SMI in assessing intestinal lesion activity in comparison with endoscopic findings in patients with CD. A detailed 1-to-1 comparison between SMI and endoscopic findings, particularly regarding ulcer size, in identical lesions evaluated by both modalities has also not yet been reported. According to the Selecting Therapeutic Targets in Inflammatory Bowel Disease (STRIDE)-II statement, the absence of ulceration may indicate endoscopic healing.^[23]^ Therefore, an approach that specifically assesses ulceration may offer a simpler and more clinically practical method for evaluating endoscopic activity in patients with CD.

This study aimed to investigate the relationship between the endoscopic findings, specifically ulcer size, and SMI findings in identical lesions of the terminal ileum, as evaluated by endoscopy and IUS, in patients with CD. We also sought to examine the sensitivity and specificity of SMI findings for predicting endoscopic ulcers.

## 2. Materials and methods

### 2.1. Participants

This retrospective cohort study included patients with CD who underwent IUS between September 2020 and April 2024 at our clinic and ileocolonoscopy for terminal ileum evaluation within 3 months before or after IUS. If IUS and ileocolonoscopy were performed multiple times within the study period, only the first examination was used for the analysis. CD diagnosis was based on the criteria determined by the Japanese Ministry of Health, Labor, and Welfare.^[24]^ We excluded patients with a history of small bowel resection and those with medical treatment changes between IUS and ileocolonoscopy.

### 2.2. Data collection

All data were obtained from the clinic’s medical records. We collected data on the following baseline characteristics: sex, age, disease duration, location-based disease type (ileitis, ileocolitis, or colitis), disease behavior, perianal disease occurrence, C-reactive protein (CRP) levels, albumin levels, and Harvey–Bradshaw index (HBI) score.^[25]^ Concomitant medications such as 5-aminosalicylic acid (5-ASA), prednisolone, budesonide, immunomodulators (azathioprine or 6-mercaptopurine), infliximab, adalimumab, ustekinumab, vedolizumab, and elemental diet therapy were also documented. Disease behavior was categorized according to the Montreal Classification as follows: nonstricturing, nonpenetrating (B1); stricturing (B2); and penetrating (B3).^[26]^

### 2.3. IUS examinations

One of 3 investigators conducted IUS examinations using Aplio a550 (Canon Medical Systems Corp., Tochigi, Japan). Small bowel scanning was initiated by defining the terminal ileum, located ventral to the psoas muscle, and then following its course proximally. The region located within 10 cm from the ileocecal valve indicated the terminal ileum. The scan was initiated using a 5 MHz convex probe, which was subsequently switched to a 9.2 MHz linear probe to obtain more detailed information. BWT was determined by measuring from the serosa–muscularis propria interface to the lumen–mucosa interface. Moreover, blood flow signals in the intestinal wall were evaluated using SMI. The parameter settings of SMI were as follows: color velocity of 1.2 cm/s and color frequency of 4 MHz.

### 2.4. IUS parameters

**Figure 1.**
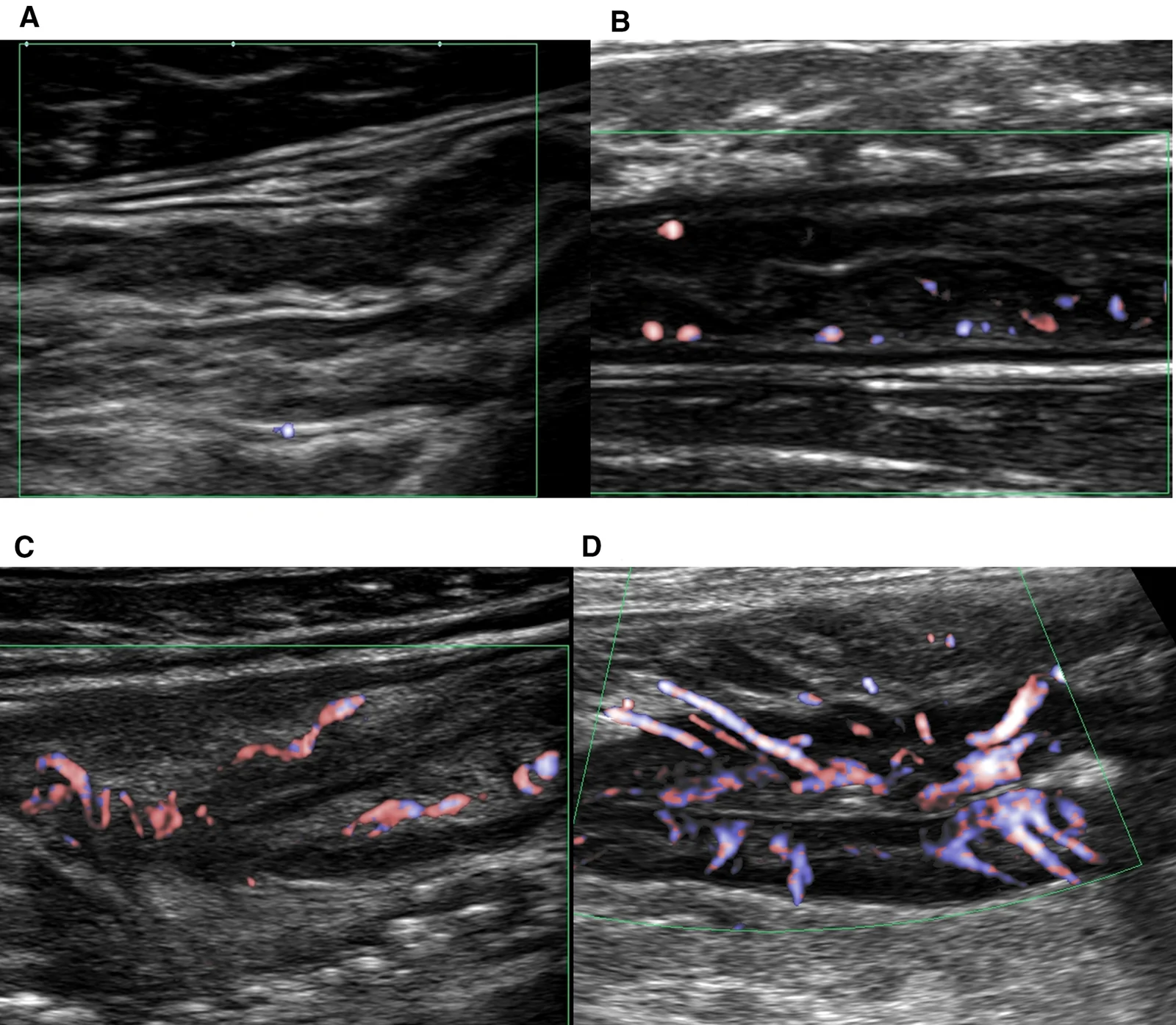
Representative examples of 4 SMI grades using intestinal ultrasonography. Grade 0: no increased vascularity (A). Grade 1: short stretches of vascularity as spots (B). Grade 2: longer stretches of vascularity (C). Grade 3: longer stretches of vascularity reaching the mesentery (D). SMI = superb microvascular imaging.

IUS findings were analyzed using 2 parameters: BWT and blood flow signals. Blood flow signals in the terminal ileum wall were evaluated using our previously reported SMI grade,^[21]^ a simplified 4-grade classification based on the Limberg score (grade 0, no increased vascularity; grade 1, short stretches of vascularity as spots; grade 2, longer stretches of vascularity; and grade 3, longer stretches of vascularity reaching the mesentery; Fig. 1). These SMI grades reflect vascularity alone, irrespective of BWT.

Conversely, we applied a modified Limberg score using superb microvascular imaging (mLS-SMI grade), replacing the blood flow component of the conventional Limberg score with the SMI grade. This mLS-SMI grade was defined as follows: grade 0, BWT < 4.0 mm; grade 1, BWT ≥ 4.0 mm without increased vascularity (SMI grade 0); grade 2, BWT ≥ 4.0 mm with short stretches of vascularity as spots (SMI grade 1); grade 3, BWT ≥ 4.0 mm with longer stretches of vascularity (SMI grade 2); and grade 4, BWT ≥ 4.0 mm with longer stretches of vascularity reaching the mesentery (SMI grade 3).

### 2.5. Ileocolonoscopic findings

Ileocolonoscopic findings were evaluated in the terminal ileum. Lesions were then classified into 4 groups according to ulcer size observed endoscopically: no ulcers, aphthous ulcers (<5 mm), small ulcers (5–20 mm), and large ulcers (>20 mm). Ulcer size was classified based on simple endoscopic score for Crohn’s disease.^[27]^ This classification was based on the ulcer’s maximal diameter as judged by the endoscopist conducting the examination.

### 2.6. Assessment of mLS-SMI and SMI grades

In this study, the primary endpoint was the sensitivity, specificity, positive predictive value (PPV), and negative predictive value (NPV) of both mLS-SMI grades and SMI grades for predicting the respective endoscopic findings. The secondary endpoint was the median BWT corresponding to the respective endoscopic findings. We also identified the proportion of the endoscopic findings in mLS-SMI grades and SMI grades. Finally, we compared the sensitivity of mLS-SMI and SMI grades for predicting the endoscopic findings.

### 2.7. Statistical analysis

We present categorical variables as frequencies and percentages, and continuous variables as medians and interquartile ranges. BWT levels stratified by respective endoscopic findings were compared using the Kruskal–Wallis test. For multiple group comparisons between respective endoscopic findings, we conducted post hoc analyses using the Dunn–Bonferroni multiple comparison test. The correlation between endoscopic findings and mLS-SMI grade or SMI grade was assessed using the χ^2^ test for linear-by-linear association and Fisher’s exact test. Using McNemar’s test, we compared the sensitivity for predicting respective endoscopic findings between the mLS-SMI and SMI grades. Receiver operating characteristic (ROC) curve analysis was performed to evaluate the diagnostic performance of SMI and mLS-SMI grades for predicting endoscopic findings. For each parameter, the area under the receiver operating characteristic curve (AUC) with a 95% confidence interval (CI) was calculated, and differences between AUCs were assessed using the DeLong method. Background factors related to endoscopic findings were evaluated using univariate and multivariate logistic regression analyses. Complete-case data were available for all variables included in both the univariate screening and subsequent multivariable logistic regression analyses; therefore, no imputation was performed and no patients were excluded because of missing data. All variables with *P*-values < 0.2 in univariate analyses were considered as variables in each multivariate logistic regression analysis. Results are presented as an odds ratio (OR) with a 95% CI. *P*-values under .05 were considered statistically significant. All statistical data were analyzed using the Statistical Package for the Social Sciences software, version 31.0 (SPSS, Chicago, IL), and EZR (Saitama Medical Center, Jichi Medical University).

### 2.8. Ethical considerations

This study obtained approval from the ethics committees of Sapporo IBD Clinic and conformed to the tenets of the Declaration of Helsinki. Given the study’s retrospective nature, written informed consent was waived. Information regarding the study was made available to patients on the institution’s website, and patients retained the right to withdraw their data at any time.

## 3. Results

### 3.1. Patient characteristics

This study included 204 patients with CD, of whom 50 (24.5%) were female. The median age was 29.0 years (interquartile range, 23.0–36.0), and the median disease duration was 5.2 years (1.9–8.9). At baseline, the median values for HBI score, CRP level, and BWT were 2 (0–4), 0.10 mg/dL (0.04–0.36), and 2.7 mm (2.1–3.6), respectively. Among the SMI grades, grade 0 was the most frequent (n = 157), while grade 3 was the least common (n = 8). Likewise, mLS-SMI grade 0 occurred most often (n = 161), and grade 4 was the least frequent (n = 5). Moreover, the majority of the patients had no ulcers (n = 96), while large ulcers were the least common (n* *= 15). Table 1 enumerates the patients’ baseline characteristics.

**Table 1:** Baseline characteristics of 204 patients with Crohn’s disease.

Variable	N = 204
Female/Male, n (%)	50 (24.5%)/154 (75.5%)
Median age, yr (IQR)	29.0 (23.0–36.0)
Median disease duration, yr (IQR)	5.2 (1.9–8.9)
Location of disease, n (%)	
Ileitis	14 (6.9%)
Ileocolitis	163 (79.9%)
Colitis	27 (13.2%)
Disease behavior, n (%)	
Nonstricturing, nonpenetrating (B1)	170 (83.3%)
Stricturing (B2)	31 (15.2%)
Penetrating (B3)	3 (1.5%)
Perianal disease, n (%)	131 (64.2%)
Harvey–Bradshaw index, median (IQR)	2 (0–4)
C-reactive protein, (mg/dL), median (IQR)	0.10 (0.04–0.36)
Albumin, (g/dL), median (IQR)	4.4 (4.1–4.6)
Concomitant medications, n (%)	
Oral 5-aminosalicylic acid	130 (63.7%)
Prednisolone	6 (2.9%)
Budesonide	3 (1.5%)
Immunomodulators	38 (18.6%)
Azathioprine	36 (17.6%)
6-mercaptopurine	2 (1.0%)
Infliximab	54 (26.5%)
Adalimumab	77 (37.7%)
Ustekinumab	5 (2.5%)
Vedolizumab	3 (1.5%)
Elemental diet therapy	27 (13.2%)
Bowel wall thickness, median (IQR)	2.7 (2.1–3.6)
Superb microvascular imaging grade, n (%)	
Grade 0	157 (77.0%)
Grade 1	27 (13.2%)
Grade 2	12 (5.9%)
Grade 3	8 (3.9%)
Modified Limberg score using superb microvascular imaging, n (%)	
Grade 0	161 (78.9%)
Grade 1	14 (6.9%)
Grade 2	13 (6.4%)
Grade 3	11 (5.4%)
Grade 4	5 (2.5%)
Endoscopic findings, n (%)	
No ulcers	96 (47.1%)
Aphthous ulcers	68 (33.3%)
Small ulcers	25 (12.3%)
Large ulcers	15 (7.4%)

IQR = interquartile range.

### 3.2. Relationship between the endoscopic findings and mLS-SMI grades

Endoscopic findings of no, aphthous, small, and large ulcers corresponded to a median BWT of 2.3, 2.8, 4.3, and 4.1 mm, respectively (Fig. S1, Supplemental Digital Content 1). Median BWT was significantly higher in small or large ulcers than in no or aphthous ulcers. Aphthous ulcers also had a significantly higher median BWT than no ulcers.

**Figure 2.**
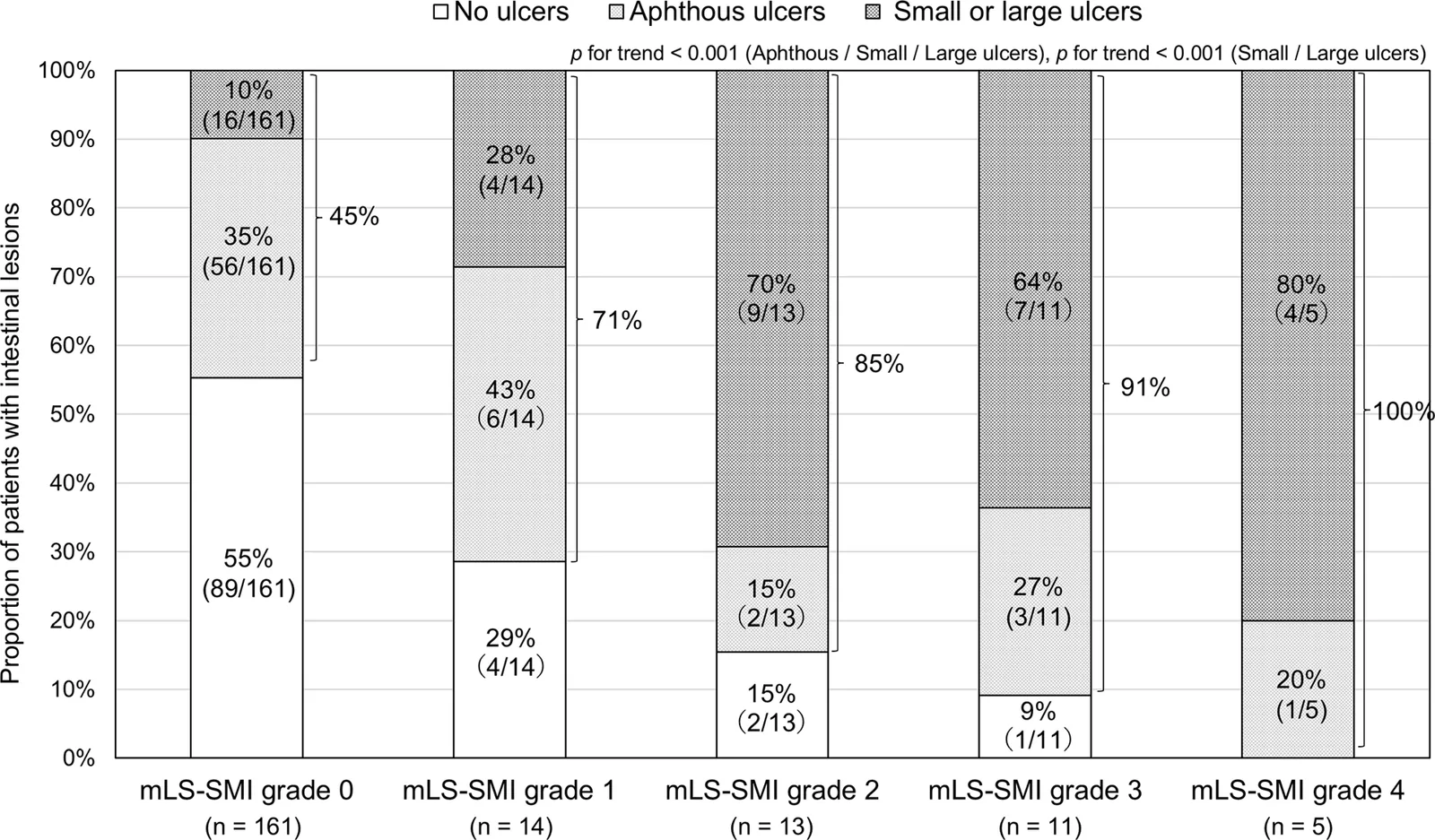
Proportions of patients with aphthous, small, or large ulcers and those with small or large ulcers stratified by mLS-SMI grades. Statistical significance was determined using the χ^2^ test for linear-by-linear association for aphthous, small, or large ulcers (*P* < .001) and for small or large ulcers (*P* < .001). mLS-SMI = modified Limberg score using superb microvascular imaging.

**Figure 3.**
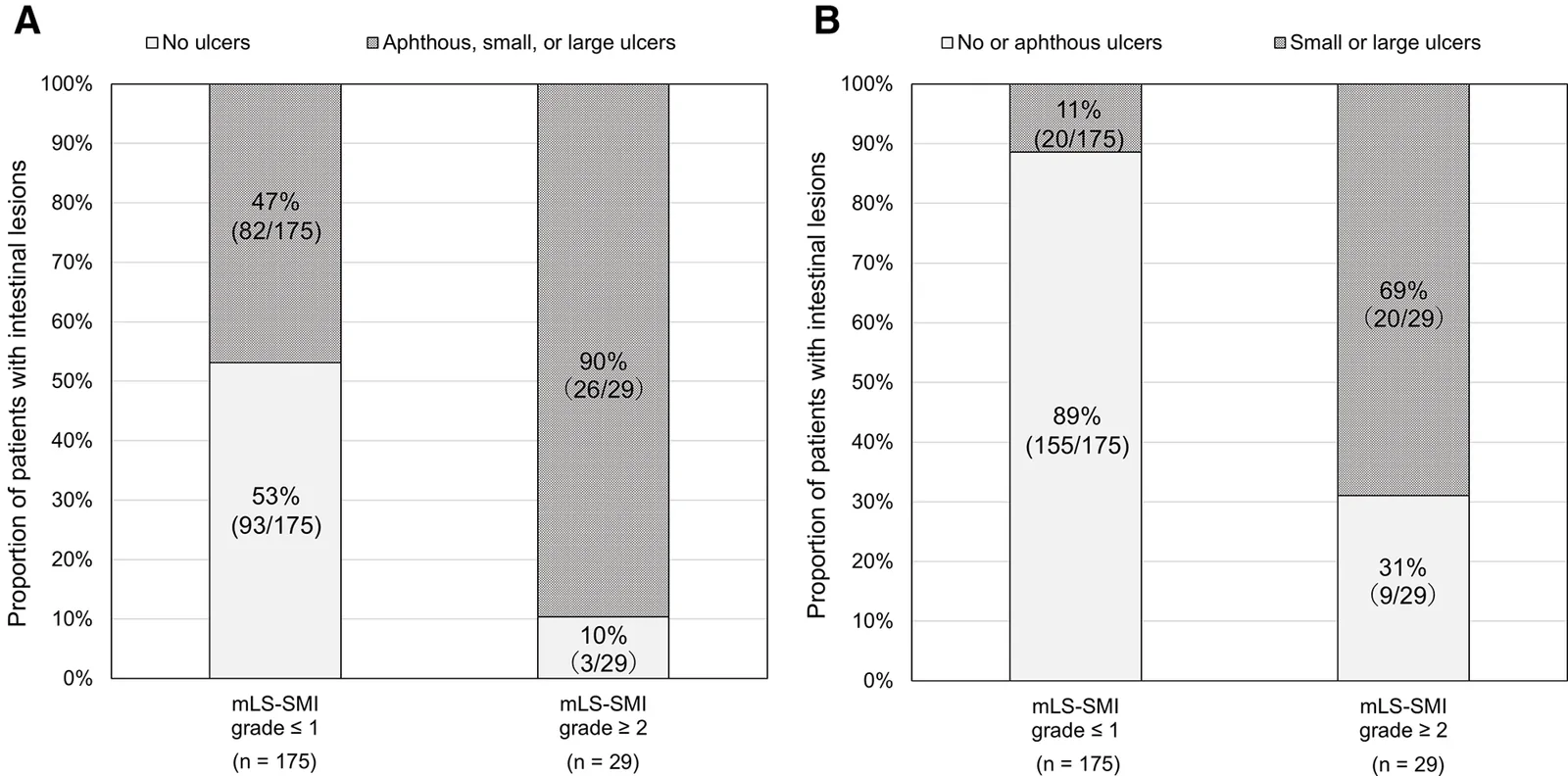
Proportion of patients with aphthous, small, or large ulcers versus those with no ulcers, stratified by mLS-SMI grade ≤ 1 or grade ≥ 2 (A). Statistical significance was determined using Fisher’s exact test (*P* < .001). Proportion of patients with small or large ulcers versus those with no ulcers or aphthous ulcers, stratified by mLS-SMI grade ≤ 1 or grade ≥ 2 (B). Statistical significance was determined using Fisher’s exact test (*P* < .001). mLS-SMI = modified Limberg score using superb microvascular imaging.

On endoscopic findings, 45%, 71%, 85%, 91%, and 100% of patients with mLS-SMI grades of 0, 1, 2, 3 and 4, respectively, had intestinal lesions such as aphthous, small, or large ulcers (Fig. 2). Additionally, 10%, 28%, 70%, 64%, and 80% of patients with mLS-SMI grades of 0, 1, 2, 3, and 4, respectively, developed small or large ulcers. As the mLS-SMI grades increased, the proportion of patients with aphthous, small, or large ulcers increased significantly (*P* < .001). The proportion of those with small or large ulcers also increased significantly as the mLS-SMI grades increased (*P* < .001). In addition, patients with mLS-SMI grade ≥ 2 more frequently had aphthous, small, or large ulcers (90% vs 47%) and small or large ulcers (69% vs 11%) than those with mLS-SMI grade ≤ 1 (*P* < .001; Fig. 3A, B).

### 3.3. Relationship between the endoscopic findings and SMI grades

**Figure 4.**
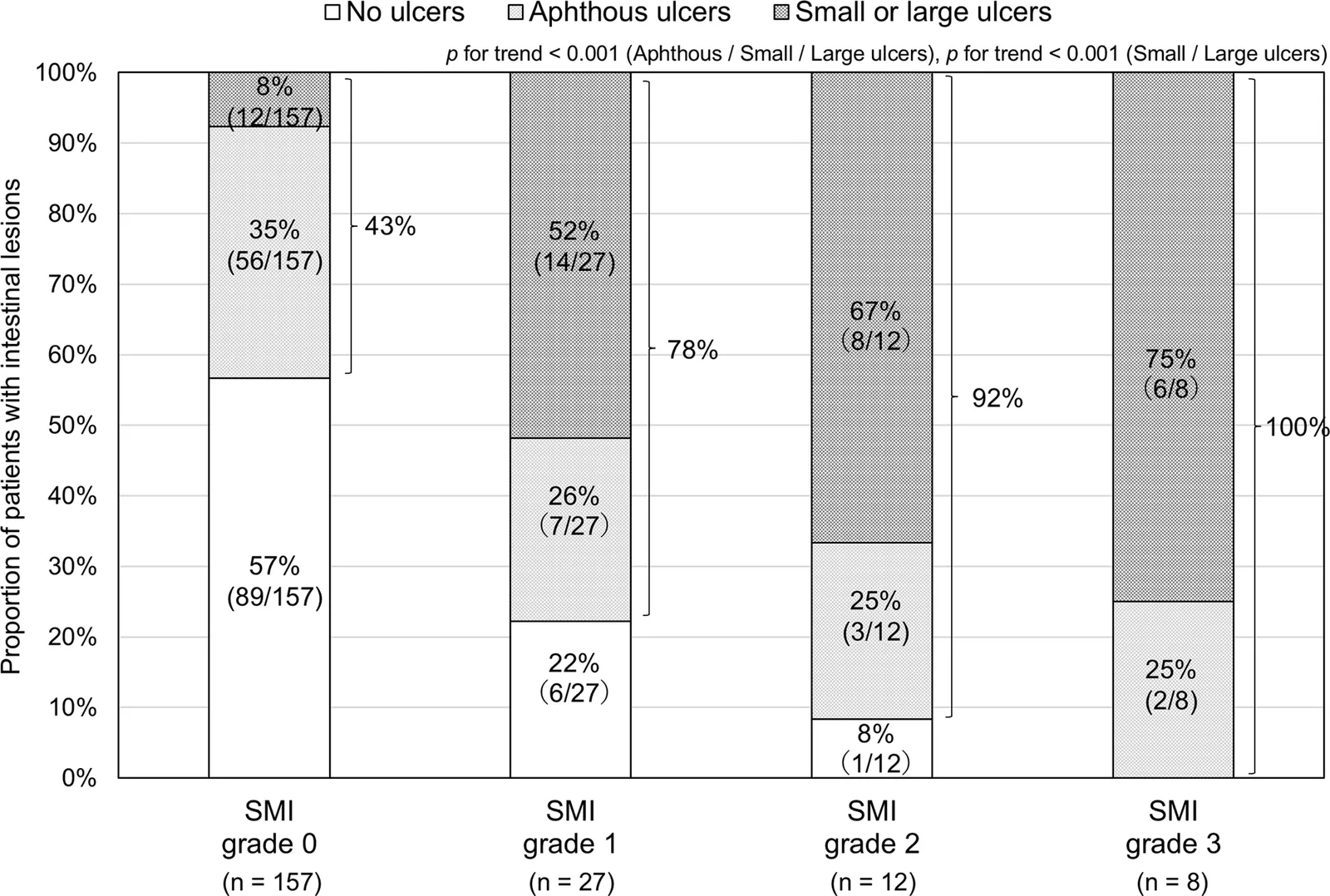
Proportions of patients with aphthous, small, or large ulcers and those with small or large ulcers stratified by SMI grades. Statistical significance was determined using the χ^2^ test for linear-by-linear association for aphthous, small, or large ulcers (*P* < .001) and for small or large ulcers (*P* < .001). SMI = superb microvascular imaging.

**Figure 5.**
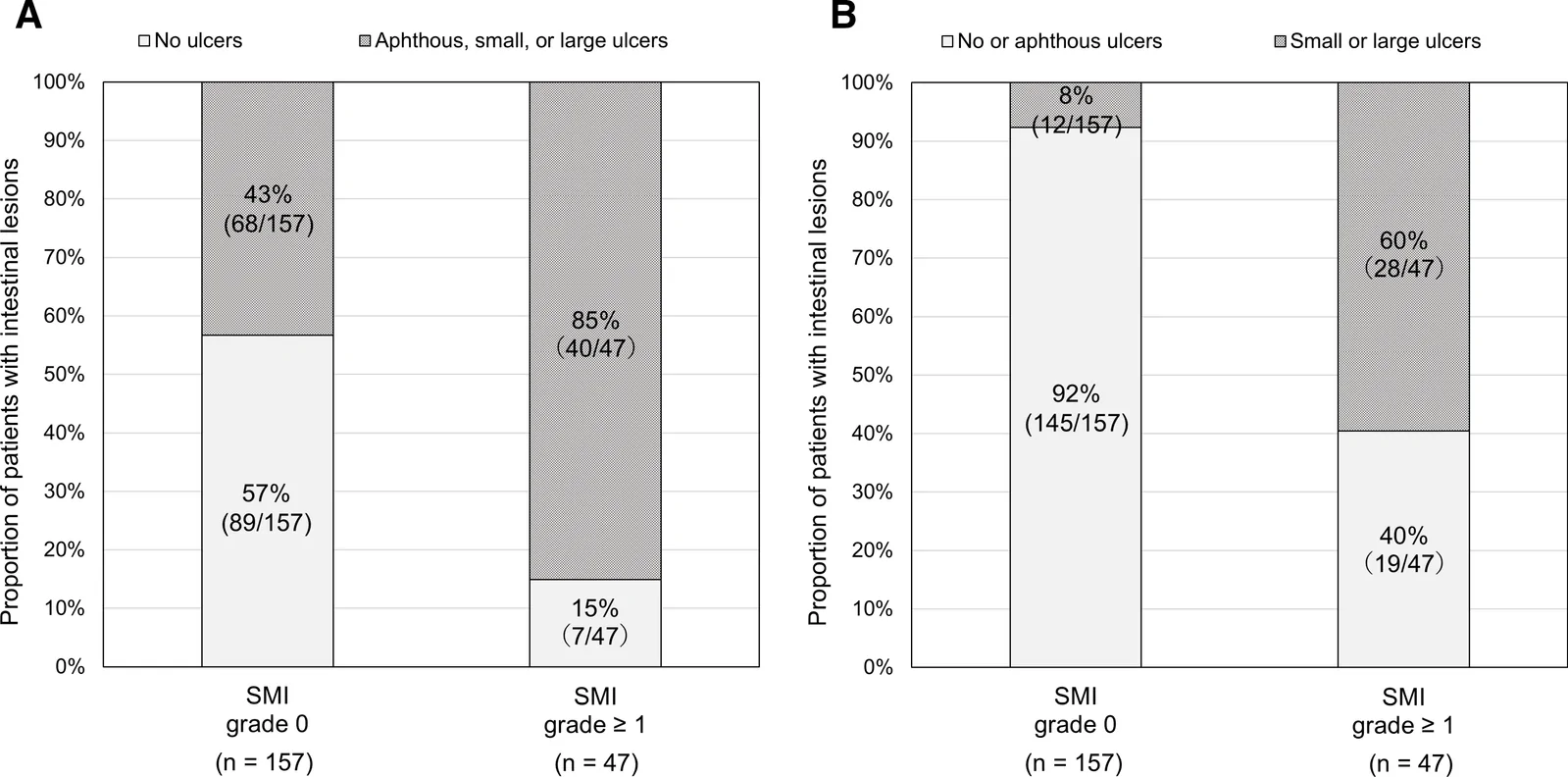
Proportion of patients with aphthous, small, or large ulcers versus those with no ulcers, stratified by SMI grade 0 or grade ≥ 1 (A). Statistical significance was determined using Fisher’s exact test (*P* < .001). Proportion of patients with small or large ulcers versus those with no ulcers or aphthous ulcers, stratified by SMI grade 0 or grade ≥ 1 (B). Statistical significance was determined using Fisher’s exact test (*P* < .001). SMI = superb microvascular imaging.

On endoscopic findings, intestinal lesions including aphthous, small, or large ulcers were observed in 43%, 78%, 92%, and 100% of patients with SMI grades of 0, 1, 2, and 3, respectively (Fig. 4). Furthermore, 8%, 52%, 67%, and 75% of patients with SMI grades of 0, 1, 2, and 3, respectively, had small or large ulcers. As the SMI grades increased, the proportion of those with aphthous, small, or large ulcers increased significantly (*P* < .001). Similarly, the proportion of patients with small or large ulcers increased significantly as the SMI grades increased (*P* < .001). Moreover, patients with SMI grade ≥ 1 more frequently had aphthous, small, or large ulcers (85% vs 43%) and small or large ulcers (60% vs 8%) than those with SMI grade 0 (*P* < .001; Fig. 5A, B). Meanwhile, 143, 14, 1, and 3 patients with BWT < 4 mm, which corresponded to mLS-SMI grade 0, had SMI grades of 0, 1, 2, and 3, respectively (Table 2). Among them, aphthous, small, and large ulcers were observed in 5, 4, and 1 patients with SMI grade 1; 0, 1, and 0 patients with SMI grade 2; and 1, 1, and 1 patients with SMI grade 3, respectively.

**Table 2:** Relationship between superb microvascular imaging grades and bowel wall thickness.

Superb microvascular imaging grade	Bowel wall thickness < 4 mm	Bowel wall thickness ≥ 4 mm	Total
Grade 0: Without increased vascularity	143 (85/50/4/4)	14 (4/6/3/1)	157 (89/56/7/5)
Grade 1: Short stretches of vascularity as spots	14 (4/5/4/1)	13 (2/2/5/4)	27 (6/7/9/5)
Grade 2: Longer stretches of vascularity	1 (0/0/1/0)	11 (1/3/4/3)	12 (1/3/5/3)
Grade 3: Longer stretches of vascularity reaching the mesentery	3 (0/1/1/1)	5 (0/1/3/1)	8 (0/2/4/2)
Total	161 (89/56/10/6)	43 (7/12/15/9)	204 (96/68/25/15)

Values in parentheses represent the number of patients with no, aphthous, small, and large ulcers, respectively.

### 3.4. Sensitivity, specificity, PPV, and NPV of the mLS-SMI and SMI grades predicting the endoscopic findings

Sensitivity, specificity, PPV, and NPV of the mLS-SMI and SMI grades for predicting endoscopic findings are summarized in Tables 3 and 4. For predicting aphthous, small, or large ulcers, the sensitivity and specificity of mLS-SMI grade ≥ 2 were 24% and 97%, respectively, whereas those of SMI grade ≥ 1 were 37% and 93% (Table 3). Accordingly, SMI grade ≥ 1 showed significantly higher sensitivity than mLS-SMI grade ≥ 2 (*P* < .001).

**Table 3:** Sensitivity, specificity, PPV, and NPV of mLS-SMI and SMI grades for predicting aphthous, small, or large ulcers.

	Sensitivity	Specificity	PPV	NPV
mLS-SMI grade ≥ 1	33% (25–43)	93% (86–97)	84% (69–93)	55% (44–63)
mLS-SMI grade ≥ 2	24% (16–33)	97% (91–99)	90% (73–98)	53% (46–61)
mLS-SMI grade ≥ 3	14% (8–22)	99% (95–100)	94% (70–100)	51% (43–58)
mLS-SMI grade ≥ 4	5% (2–10)	100% (96–100)	100% (48–100)	48% (8–22)
SMI grade ≥ 1	37% (28–47)	93% (86–97)	85% (72–94)	57% (49–65)
SMI grade ≥ 2	18% (11–26)	99% (95–100)	95% (75–100)	52% (44–59)
SMI grade ≥ 3	7% (3–14)	100% (96–100)	100% (63–100)	49% (42–56)

mLS = modified Limberg score, NPV = negative predictive value, PPV = positive predictive value, SMI = superb microvascular imaging.

**Table 4:** Sensitivity, specificity, PPV, and NPV of mLS-SMI and SMI grades for predicting small or large ulcers.

	Sensitivity	Specificity	PPV	NPV
mLS-SMI grade ≥ 1	60% (43–75)	88% (82–93)	56% (41–70)	90% (85–94)
mLS-SMI grade ≥ 2	50% (34–66)	95% (90–98)	69% (49–85)	89% (83–93)
mLS-SMI grade ≥ 3	28% (15–44)	97% (93–99)	69% (41–89)	85% (79–90)
mLS-SMI grade ≥ 4	10% (3–24)	99% (97–100)	80% (28–100)	82% (76–87)
SMI grade ≥ 1	70% (54–83)	88% (82–93)	60% (44–74)	92% (87–96)
SMI grade ≥ 2	35% (21–52)	96% (92–99)	70% (46–88)	86% (80–91)
SMI grade ≥ 3	15% (6–30)	99% (96–100)	75% (35–97)	83% (77–88)

mLS = modified Limberg score, NPV = negative predictive value, PPV = positive predictive value, SMI = superb microvascular imaging.

For predicting small or large ulcers, the sensitivity and specificity of mLS-SMI grade ≥ 2 were 50% and 95%, respectively, while those of SMI grade ≥ 1 were 70% and 88% (Table 4). Similarly, SMI grade ≥ 1 demonstrated significantly higher sensitivity than mLS-SMI grade ≥ 2 (*P* = .008).

### 3.5. Logistic regression analyses for predicting endoscopic findings

Univariable logistic regression analysis identified age, disease duration, HBI, CRP, disease location (ileocolitis type), BWT, SMI grade, and concomitant biologic use as factors associated with the presence of aphthous, small, or large ulcers. In multivariable logistic regression analysis, age (OR 0.950, *P* = .017), BWT (OR 1.914, *P* < .001), SMI grade (OR 2.304, *P* = .036), and concomitant biologic use (OR 0.264, *P* < .001) remained independently associated with the presence of aphthous, small, or large ulcers (Table 5). For predicting small or large ulcers, univariable logistic regression analysis identified disease location (ileocolitis type), disease behavior (B2/B3), BWT, and SMI grade as significant factors. In multivariable logistic regression analysis, BWT (OR 2.059, *P* < .001) and SMI grade (OR 2.425, *P* = .002) remained independently associated with the presence of small or large ulcers (Table 6).

**Table 5:** Univariable and multivariable logistic regression analyses for endoscopic aphthous, small, or large ulcers.

Variables	Univariate analysis			Multivariate analysis		
	Odd’s ratio	95% CI	*P*-value	Odd’s ratio	95% CI	*P*-value
Sex (F)	1.058	0.558–2.006	.863			
Age	0.966	0.937–0.995	.023	0.950	0.911–0.991	.017
Disease duration	0.957	0.913–1.003	.068	1.022	0.959–1.089	.508
Harvey–Bradshaw index	1.102	0.995–1.221	.063	1.019	0.896–1.159	.776
C-reactive protein	1.574	0.972–2.550	.065	1.099	0.727–1.663	.654
Location of disease (Ileocolitis)	2.025	1.006–4.076	.048	1.676	0.710–3.959	.239
Disease behavior (B2/B3)	0.868	0.416–1.814	.707			
Perianal disease	1.151	0.649–2.043	.630			
BWT	2.048	1.537–2.728	<.001	1.914	1.326–2.762	<.001
SMI grade	4.436	2.183–9.013	<.001	2.304	1.057–5.023	.036
Concomitant biologic use	0.311	0.164–0.589	<.001	0.264	0.123–0.565	<.001

BWT = bowel wall thickness, CI = confidence interval, SMI = superb microvascular imaging.

**Table 6:** Univariable and multivariable logistic regression analyses for endoscopic small or large ulcers.

Variables	Univariate analysis			Multivariate analysis		
	Odd’s ratio	95% CI	*P*-value	Odd’s ratio	95% CI	*P*-value
Sex (F)	1.216	0.556–2.656	.624			
Age	1.013	0.979–1.048	.455			
Disease duration	1.003	0.948–1.061	.930			
Harvey–Bradshaw index	1.055	0.937–1.188	.374			
C-reactive protein	1.198	0.896–1.602	.224			
Location of disease (Ileocolitis)	1.969	0.719–5.391	.188	2.381	0.705–8.038	.162
Disease behavior (B2/B3)	2.766	1.228–6.229	.014	1.998	0.691–5.775	.201
Perianal disease	1.382	0.655–2.916	.396			
BWT	2.683	1.938–3.715	<.001	2.059	1.392–3.044	<.001
SMI grade	4.724	2.808–7.946	<.001	2.425	1.383–4.252	.002
Concomitant biologic use	1.473	0.671–3.233	.335			

BWT = bowel wall thickness, CI = confidence interval, SMI = superb microvascular imaging.

### 3.6. ROC curve analysis

**Figure 6.**
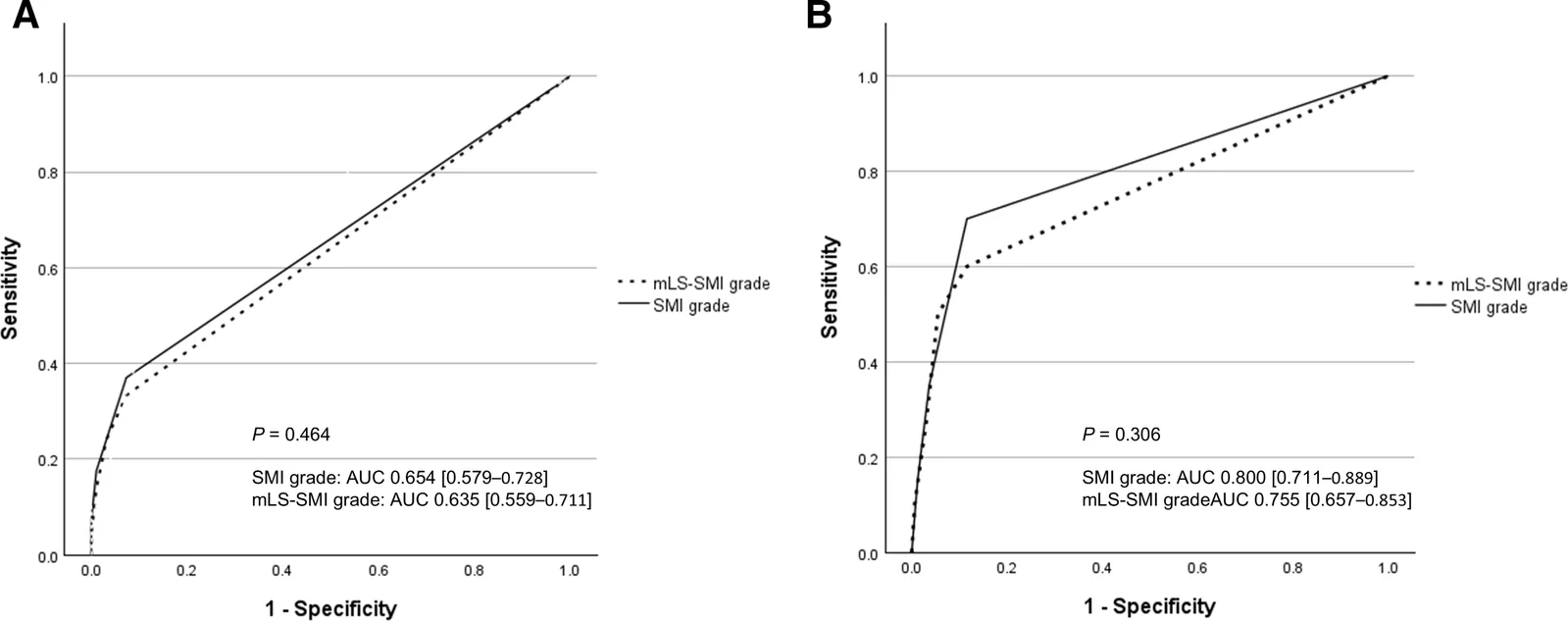
Receiver operating characteristic curves of SMI and mLS-SMI grades for predicting aphthous, small, or large ulcers (A) and small or large ulcers (B). AUCs were compared using the DeLong method (A: *P* = .464; B: *P* = .306). AUC = area under the receiver operating characteristic curve, mLS-SMI = modified Limberg score using superb microvascular imaging, SMI = superb microvascular imaging.

ROC curve analysis was performed to evaluate the diagnostic performance of SMI and mLS-SMI grades for predicting endoscopic findings (Fig. 6). For predicting aphthous, small, or large ulcers, the AUCs were 0.654 (95% CI, 0.579–0.728) for SMI grade and 0.635 (95% CI, 0.559–0.711) for mLS-SMI grade, without significant differences between the 2 (DeLong test, *P* = .464). For predicting small or large ulcers, the AUCs were 0.800 (95% CI, 0.711–0.889) for SMI grade and 0.755 (95% CI, 0.657–0.853) for mLS-SMI grade, similarly showing no significant differences (DeLong test, *P* = .306).

## 4. Discussion

To the best of our knowledge, this is the largest cohort study evaluating intestinal lesion activity in patients with CD using SMI and the first to investigate the relationship between SMI-detected blood flow signals and endoscopic findings focused on ulcer size. In this study, increasing mLS-SMI and SMI grades were associated with a higher proportion of patients with aphthous, small, or large ulcers and with small or large ulcers. In addition, SMI grade remained independently associated with the presence of endoscopic ulcers even after adjustment for BWT and other clinical variables. Furthermore, SMI grade ≥ 1 showed numerically higher sensitivity than mLS-SMI grade ≥ 2 for predicting small or large ulcers.

Recently, 4 different IUS scoring tools have been proposed to assess inflammatory bowel activity: International Bowel Ultrasound Segmental Activity Score (IBUS-SAS),^[28]^ Bowel Ultrasound Score,^[29]^ Simple Ultrasound Score,^[30]^ and Simple Ultrasound Score for Crohn’s Disease.^[31]^ IBUS-SAS uses 4 IUS parameters: BWT, color Doppler signals (CDS), BWS, and inflamed mesentery fat (i-fat). The 3 other IUS scores utilize only BWT and CDS. Dragoni et al compared these 4 IUS scores in terms of their correlation with endoscopic activity in 73 patients with CD.^[32]^ They found that IBUS-SAS showed the strongest correlation with endoscopic and clinical activity. However, this tool is not available in centers with limited IUS experience, considering that interpreting BWS and i-fat is more complex and requires additional training. An international panel of experts has suggested definitions of treatment response and remission for IUS according to BWT and CDS parameters.^[33]^ Therefore, only BWT is used in simpler scores, while CDS may be desirable for the widespread use of IUS for evaluating bowel activity in CD.

BWT is the most established parameter for assessing disease activity in CD, with low interobserver variability. Meta-analysis revealed that, for detecting active lesions of established CD, a cutoff value of 3 mm provided a sensitivity and specificity of 89% and 96%, while other cutoff values (≥4 mm) yielded 87% and 98%, respectively.^[28]^ In our study, a BWT ≥ 4 mm without increased vascularity defined mLS-SMI grade ≥ 1; therefore, the sensitivity and specificity of BWT ≥ 4 mm were identical to those of mLS-SMI grade ≥ 1. For predicting small or large ulcers, the sensitivity and specificity of mLS-SMI grade ≥ 1 were 60% and 88%, respectively, which were relatively lower than those reported in Dong et al’s meta-analysis.^[34]^ The reason underlying this discrepancy remains uncertain. However, this meta-analysis revealed that the reference standard used to assess CD activity varied across studies. Some studies used small bowel follow-through to evaluate active disease, whereas others relied on postoperative anastomotic recurrence as an indicator of CD activity. These methodological differences suggest that the meta-analysis included more advanced lesions overall. Conversely, our study defined active disease more strictly according to endoscopic ulcer size, likely encompassing relatively mild lesions. This difference in disease spectrum may explain the sensitivity discrepancies between the meta-analysis and our results.

The bowel wall’s vascularity is also an important parameter of IUS for assessing intestinal activities in CD. In general, vascularity and blood flow are assessed using color Doppler methods. However, these methods can assess only larger vessels with signals above the noise level and with flow velocities above the threshold of the wall filter, which is used to suppress tissue motion. Conventional color Doppler methods cannot visualize very slow low-volume blood flow.^[12]^ To resolve these limitations, some European and Asian countries use CEUS. CEUS accurately depicts bowel wall perfusion and the perienteric tissues following the intravenous administration of microbubble contrast agents. Two meta-analyses found CEUS to be a highly sensitive, accurate, and specific method for assessing inflammatory bowel disease activity. However, of note, using CEUS in bowel assessment is currently off-label in most countries.^[16]^ The recently developed SMI technique has superior sensitivity, especially for detecting slow blood flow, compared with conventional color Doppler ultrasound.^[15-17]^ Additionally, SMI does not require contrast agents and is thereby cost-effective and free from safety concerns related to contrast administration.

The conventional Limberg score, the most widely used scoring tool for the semiquantitative grading of bowel wall vascularity, is based on BWT (<4 or ≥4 mm) combined with the size and extent of the color or power Doppler signals.^[9]^ A Limberg score of ≥2 is considered abnormal and consistent with active inflammation. Importantly, in our study, multivariable logistic regression analysis demonstrated that SMI grade remained independently associated with endoscopic ulcers even after adjusting for BWT. Indeed, a substantial proportion of patients with positive SMI findings had a BWT < 4 mm, suggesting that SMI may detect inflammatory activity even in lesions without marked bowel wall thickening. These findings suggest that assessing bowel wall vascularity with SMI may provide additional information beyond BWT alone. However, in the ROC analysis, despite the higher AUC of the SMI grade than that of the mLS-SMI grade, the difference was not significant. In addition, while SMI grade ≥ 1 showed significantly higher sensitivity than mLS-SMI grade ≥ 2 for detecting small or large ulcers, the sensitivity remained only moderate (~70%). Moreover, the small number of patients classified as the highest SMI and mLS-SMI grades limited the precision of the corresponding diagnostic performance estimates, as reflected by the wide CIs. Together, our results suggest that SMI may be a useful complementary parameter for evaluating intestinal inflammatory activity in CD. Assessing bowel wall vascularity using SMI may provide additional information on intestinal inflammation, particularly in cases where BWT alone may underestimate disease activity. Nonetheless, our study found that the sensitivity for identifying aphthous, small, or large ulcers was comparatively low, suggesting that SMI may face challenges in reliably detecting aphthous ulcers.

One strength of our study is that it represents the largest cohort to assess the endoscopic findings of intestinal lesions in patients with CD through SMI and that it is the first to investigate the relationship between blood flow signals detected by SMI and endoscopic findings focused on ulcer size in patients with CD. However, given the retrospective observational design of this study conducted at a single center, several limitations should be considered. First, ileocolonoscopy was performed within 3 months before or after IUS, which may have introduced temporal discrepancies between the 2 examinations. Second, ulcer size evaluation on endoscopy relied on visual estimation by the endoscopist and was not based on objective measurements. Third, interobserver variability in SMI grading across the 3 IUS operators was not formally assessed. Therefore, the findings of this study should be interpreted cautiously. Fourth, a priori sample size calculation was not performed because this study was based on a retrospective analysis of consecutively collected clinical data. Fifth, we did not compare SMI with color or power Doppler ultrasound. Finally, this study cannot ensure precise correspondence between IUS and endoscopic locations. To mitigate this issue, we restricted our evaluation to the terminal ileum, which can be most consistently assessed by both modalities. However, this restriction may limit the generalizability of our findings to other intestinal segments. Additionally, no formal adjustment for multiple comparisons was performed because the evaluated diagnostic thresholds were predefined and clinically related. Nevertheless, data on the outcomes obtained in this study can be helpful for IUS using SMI applied in patients with CD. As ultrasound technology continues to advance, including the development of novel modalities such as SMI, previous evidence may need to be updated to reflect these technological improvements. Future prospective multicenter studies should formally evaluate both interobserver and intraobserver reliability of SMI grading to further validate its reproducibility and facilitate its broader clinical implementation.

## 5. Conclusions

The proportion of patients with endoscopic findings, including aphthous, small, or large ulcers, in the terminal ileum increased as the mLS-SMI and SMI grades increased. The sensitivity for predicting small or large ulcers was significantly higher in SMI grade ≥ 1 than in mLS-SMI grade ≥ 2. Therefore, bowel vascularity assessment using SMI within IUS may be useful for identifying active ulcerative lesions in patients with CD. Furthermore, SMI grade may provide additional information, particularly in cases where bowel wall thickening is not apparent when evaluating small or large ulcers in the terminal ileum.

## Acknowledgements

The authors would like to thank all patients enrolled in our study. We would also like to thank Enago (www.enago.jp) for the English language review.

## Author contributions

**Conceptualization:** Masanao Nasuno, Maki Miyakawa, Hiroki Tanaka.

**Data curation:** Masanao Nasuno, Airi Konno, Kazuki Shimoyama, Takahito Hamada, Kohei Sugiyama, Maki Miyakawa, Hiroki Tanaka.

**Formal analysis:** Masanao Nasuno, Maki Miyakawa, Masanori Nojima, Hiroki Tanaka.

**Investigation:** Masanao Nasuno, Airi Konno, Kazuki Shimoyama, Takahito Hamada, Maki Miyakawa.

**Supervision:** Hiroki Tanaka.

**Writing – original draft:** Masanao Nasuno, Maki Miyakawa, Hiroki Tanaka.

**Writing – review & editing:** Masanao Nasuno, Airi Konno, Kohei Sugiyama, Maki Miyakawa, Hiroki Tanaka.


